# Supplementary material for: Admixture-enabled selection for rapid adaptive evolution in the Americas
Source: Genome Biol. 2020 Feb 7;21:29. doi: 10.1186/s13059-020-1946-2 (PMC7006128; doi:10.1186/s13059-020-1946-2)
Supplement: Supplementary file 1 — Additional file 1:Figures S1-S11, Tables S2-S3, and Supplementary Methods. [file 13059_2020_1946_MOESM1_ESM.pdf]

Supplementary Information for

Admixture-enabled selection for rapid adaptive evolution in the Americas

Emily T. Norris, Lavanya Rishishwar, Aroon T. Chande, Andrew B. Conley, Kaixiong Ye, Augusto Valderrama-Aguirre, and I. King Jordan

Figure S1. Correspondence between continental ancestry estimates for LA populations generated by ADMIXTURE (x-axis) and RFMix (y-axis). 2

Figure S2. Correspondence between local ancestry estimates for LA populations generated by RFMix using a variable recombination rate (x-axis) and a uniform recombination rate (y-axis) for chromosome 6. 3

Figure S3. Correspondence between Native American ancestry estimates for LA populations generated by using Native American reference populations (x-axis) versus East Asian reference populations (y-axis). 4

Figure S4. Comparison of global versus local continental ancestry inference for two admixed individuals. 5

Figure S5. Scheme of the ancestry enrichment analysis method used for this study. 6

Figure S6. Observed versus expected ancestry enrichment across the four LA populations studied here. 7

Figure S7. Ancestry enrichment power analysis. 8

Figure S8. African ancestry enrichment power analysis. 9

Figure S9. Observed versus expected ancestry enrichment across the four LA populations studied here for chromosome 6. 10

Modeling admixture-enabled selection at single loci 11

Figure S10. Modelling the strength of admixture-enabled selection at the MHC locus. 12

Table S2. Human populations analyzed as part of this study. 13

Polygenic ancestry enrichment 14

Table S3. Sources of the polygenic trait gene sets analyzed as part of this study. 14

Figure S11. Simulation of random polygenic trait gene sets used to compute statistical significance of polygenic ancestry enrichment (PAE). 15

References 16

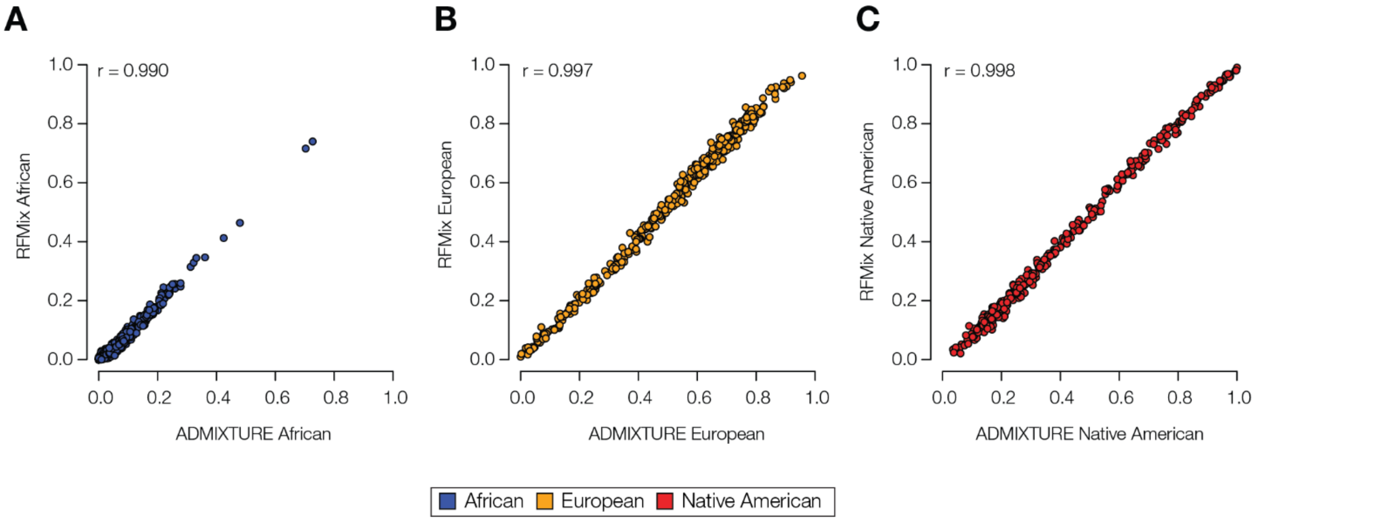

Figure S1. **Correspondence between continental ancestry estimates for LA populations generated by ADMIXTURE (x-axis) and RFMix (y-axis).** Ancestry estimates generated for individuals from the four LA populations studied here – Colombia, Mexico, Peru, and Puerto Rico – are shown separately for (A) African, (B) European, and (C) Native American ancestry. Correlation between ancestry estimates generated using the two programs are measured by Pearson's correlation coefficient (r).

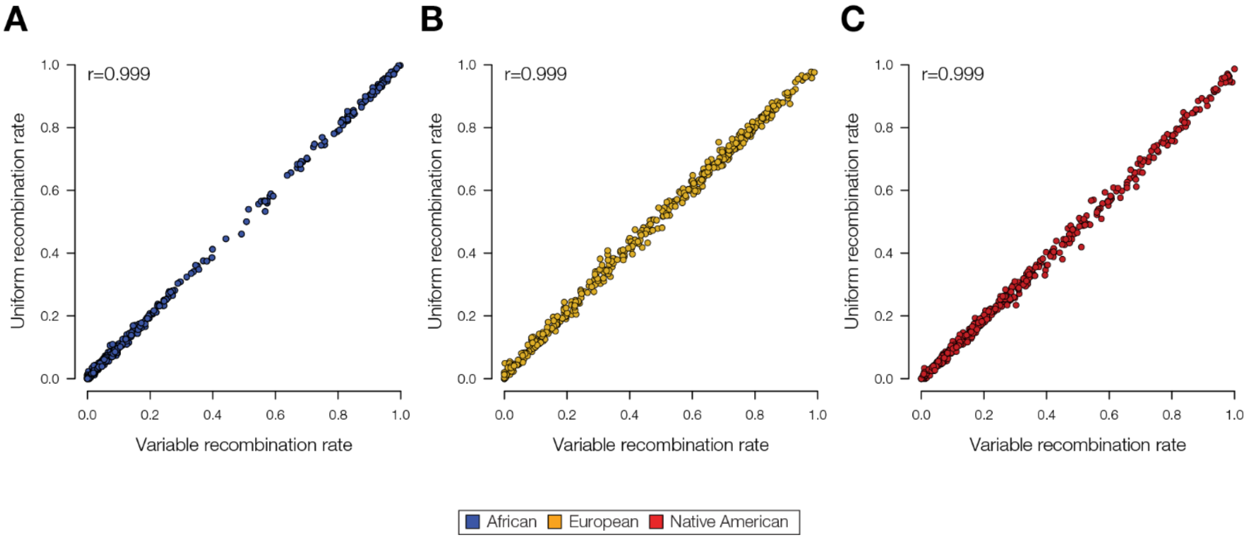

Figure S2. **Correspondence between local ancestry estimates for LA populations generated by RFMix using a variable recombination rate (x-axis) and a uniform recombination rate (y-axis) for chromosome 6.** Ancestry estimates generated for individuals from the four LA populations studied here – Colombia, Mexico, Peru, and Puerto Rico – are shown separately for (A) African, (B) European, and (C) Native American ancestry. Correlation between ancestry estimates generated using the two sets of reference populations are measured by Pearson's correlation coefficient (r).

It may be the case that modern Native American samples used as proxy ancestral source populations are not closely related to the actual ancestral populations that admixed to form modern LA populations. This could be particularly true for Puerto Rico, where the almost all of the native Taíno inhabitants were killed within the first 100 years of Spanish contact. Nevertheless, since we are assigning local ancestry for continental ancestry groups that are very diverged – African, European, and Native American – it should not matter if the exact same Native American reference populations are not used to infer local ancestry since the Native American segments in the admixed populations will be far more closely related to reference Native American populations that to reference African or European populations. We validated this assumption by using East Asian reference populations (CHB and JPT from the 1000 Genomes Project) as a surrogate for Native American reference populations for local ancestry assignment with RFMix.

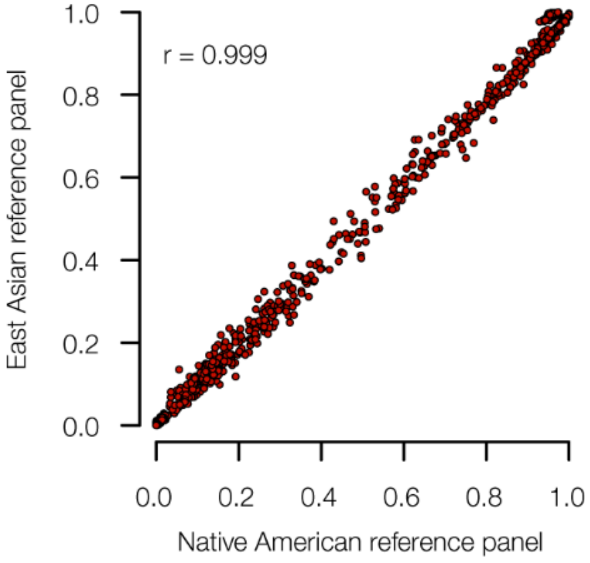

Figure S3. **Correspondence between Native American ancestry estimates for LA populations generated by using Native American reference populations (x-axis) versus East Asian reference populations (y-axis).** Native American ancestry estimates are shown for individuals from the four LA populations studied here – Colombia, Mexico, Peru, and Puerto Rico. Correlation between ancestry estimates generated using the two sets of reference populations are measured by Pearson’s correlation coefficient (r).

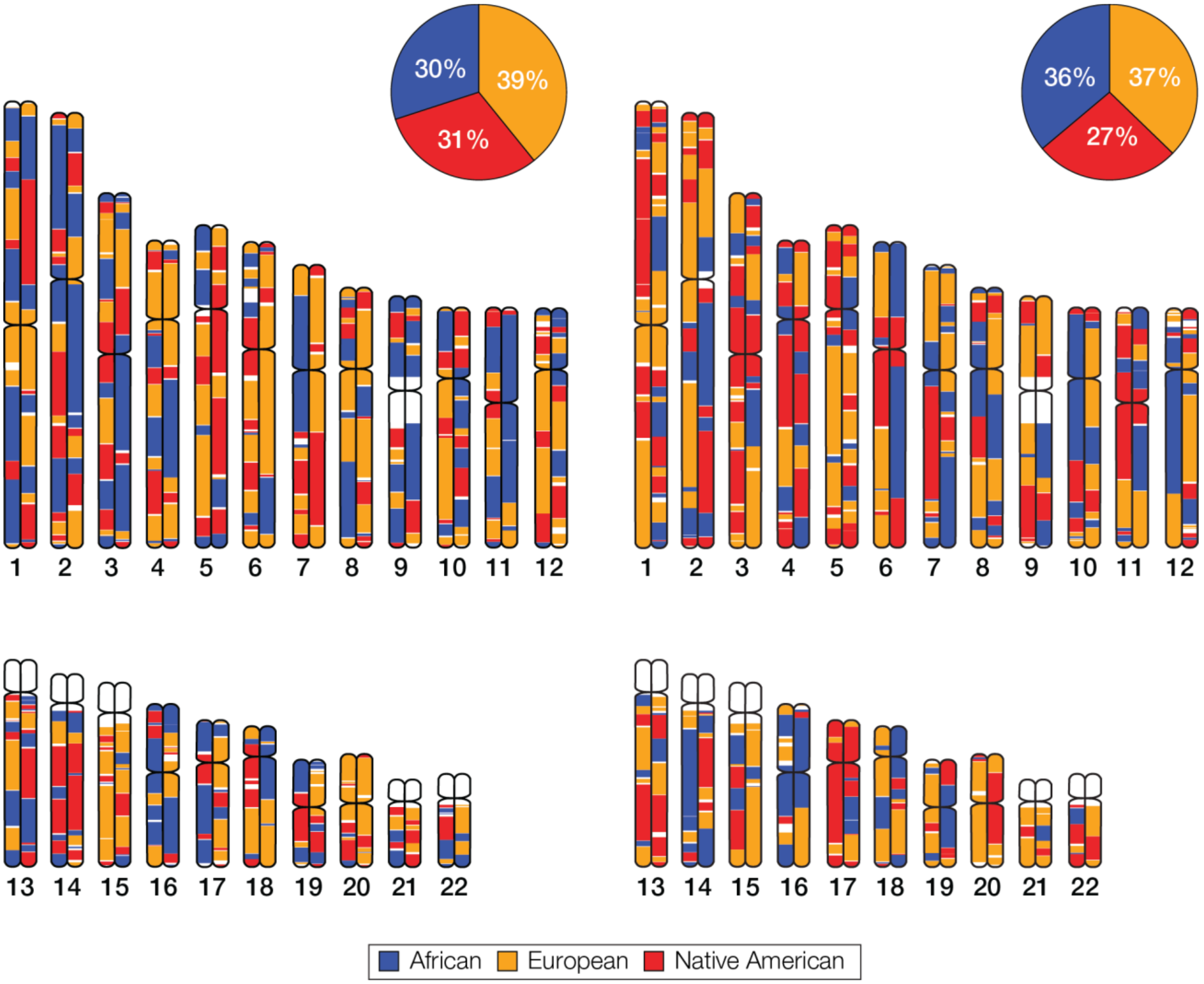

Figure S4. **Comparison of global versus local continental ancestry inference for two admixed individuals.** Continental ancestry estimates – for African (blue), European (orange), and Native American (red) ancestry components – were inferred using global (ADMIXTURE) and local (RFMix) ancestry methods. Global ancestry estimates are shown as pie charts, and local ancestry estimates (i.e. ancestry-specific haplotype assignments) are shown as chromosome ideograms. These two individuals have very similar levels of global ancestry but highly distinct local ancestry patterns.

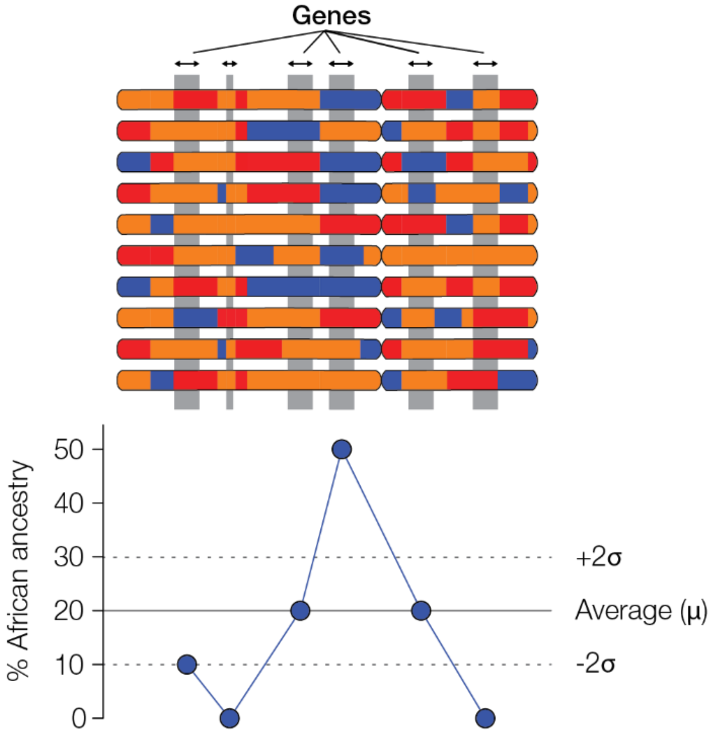

Figure S5. **Scheme of the ancestry enrichment analysis method used for this study.** Ten haploid chromosomes from a population are shown with corresponding regions aligned and ancestry-specific haplotypes indicated (as in Figure S2). Gene-specific ancestry enrichment (z_{anc}) is expressed as the number of standard deviations above or below the genome-wide ancestry fraction for the population.

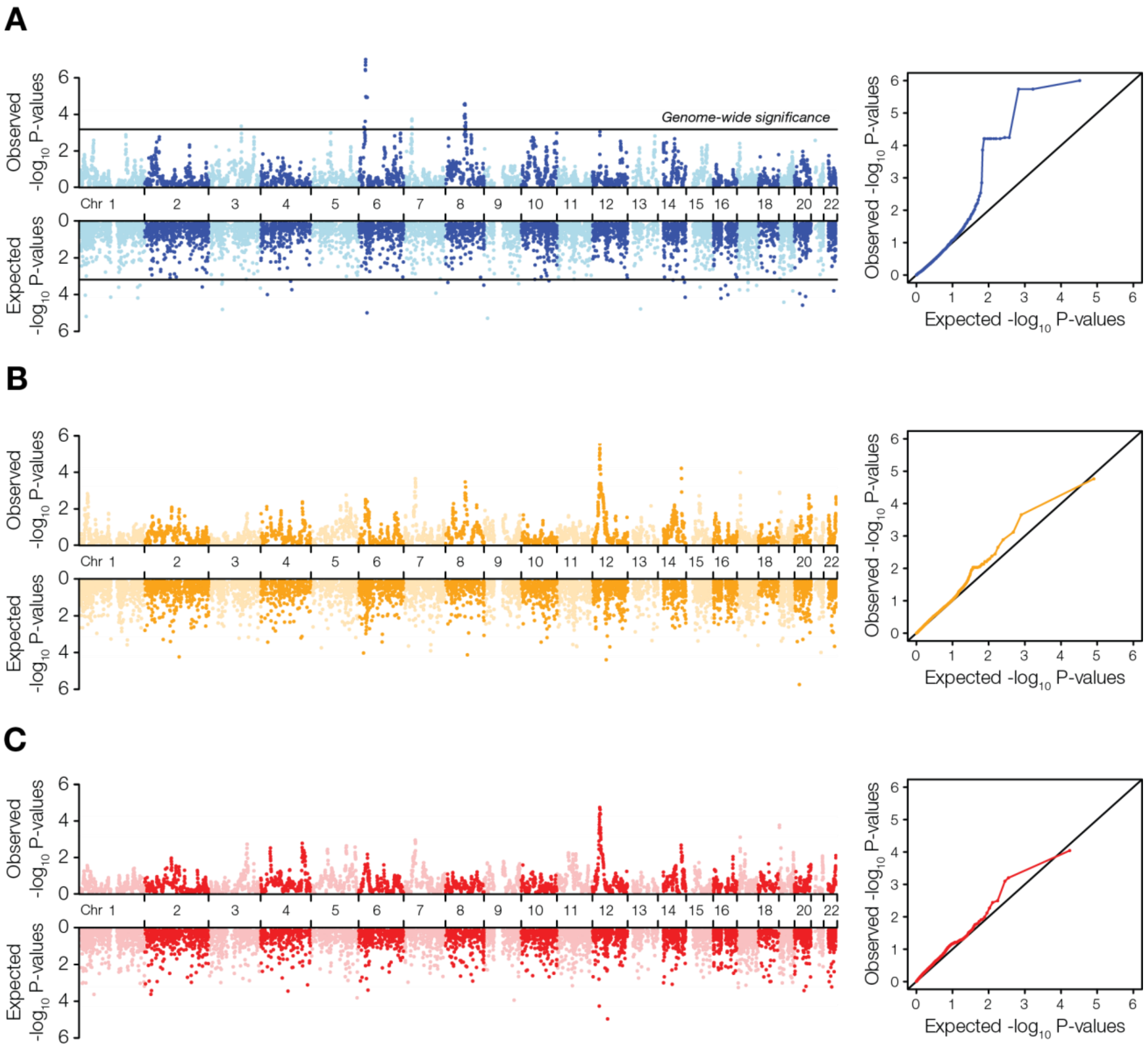

Figure S6. **Observed versus expected ancestry enrichment across the four LA populations studied here.** Manhattan plots and QQ plots show the genome-wide distributions of observed versus expected ancestry enrichment $-\log_{10} P$ values for African (A), European (B), and Native American (C) ancestry components. P-values correspond to the combined cross-population ancestry enrichment values (F_{CS}), calculated as described in the manuscript. Expected values were generated based on size matched simulated admixed populations, as described in the Methods section. On the African ancestry Manhattan plot, lines indicate genome-wide statistically significant cross-population ancestry enrichment (FDR $q < 0.05$). There were no genome-wide significant cross-population ancestry enrichment peaks for the European and Native American ancestry components. Ancestry-specific QQ plots also illustrate the deviation of observed versus expected ancestry enrichment for the African component, and the similarity of observed versus expected ancestry enrichment for the European and Native American components.

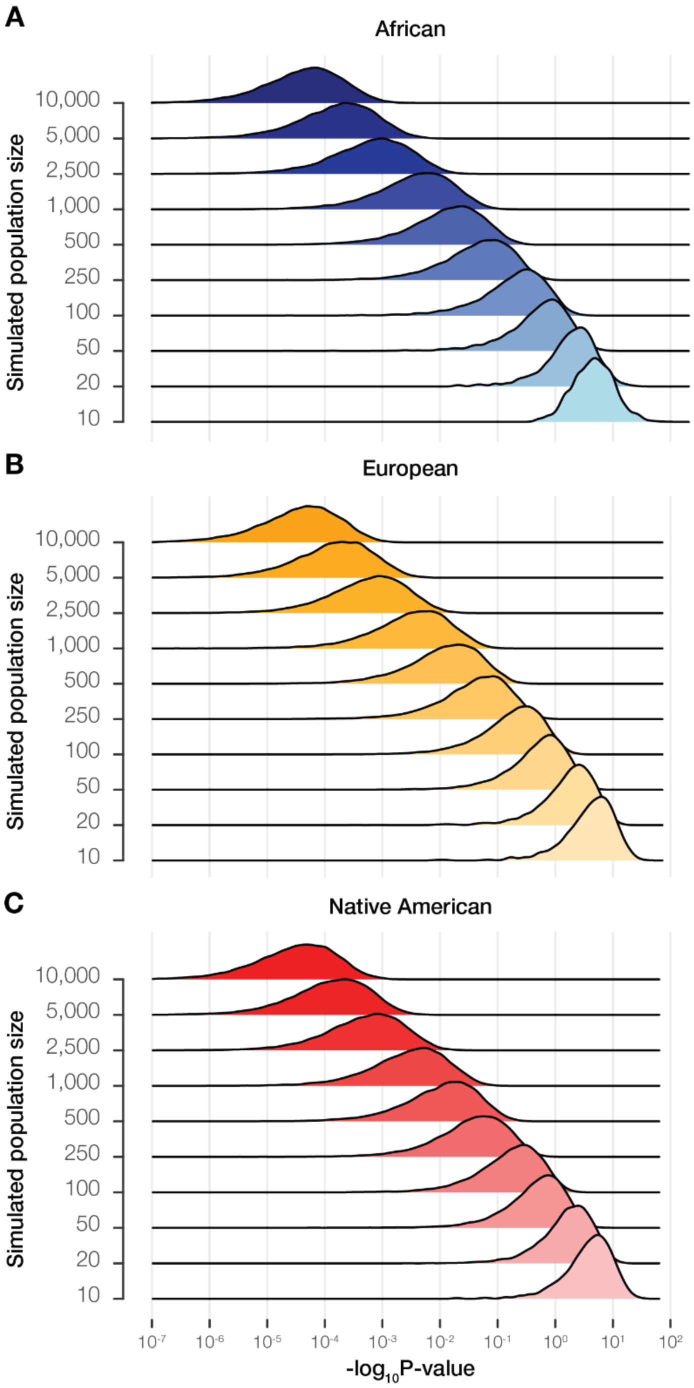

Figure S7. **Ancestry enrichment power analysis.** Four admixed LA populations were simulated across a range of population sizes (see Methods). Distributions of $-\log_{10} P$ values corresponding to combined cross-population ancestry enrichment values (F_{CS}) are shown across the range of simulated population sizes for each ancestry component: African (A), European (B), and Native American (C). This analysis was used as an additional, non-parametric method to compute the probability of observing ancestry-specific F_{CS} by chance alone, at different population sizes.

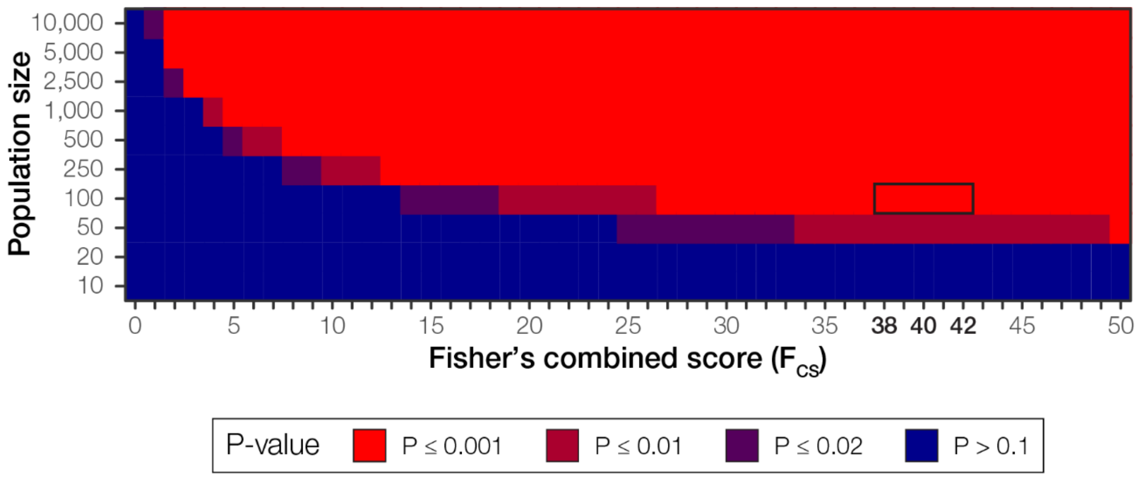

Figure S8. **African ancestry enrichment power analysis.** Four admixed LA populations were simulated across a range of population sizes (see Methods). Cross-population African enrichment values (F_{CS}), and their corresponding P values, were computed across the range of population sizes (see Figure S5). The plot shows the relationship of F_{CS} and P-values for different population sizes, *i.e.* the relationship between population size and the power of the cross-population ancestry enrichment metric. For African ancestry, a statistically significant FDR $q < 0.05$ corresponds to P<0.001 (bright red). Significant P values are more common at higher sample sizes. The observed African F_{CS} values at the MHC locus range from 38-42 (see boxed cells), which are well powered to detect significant ancestry enrichment at population sizes of ~100 individuals.

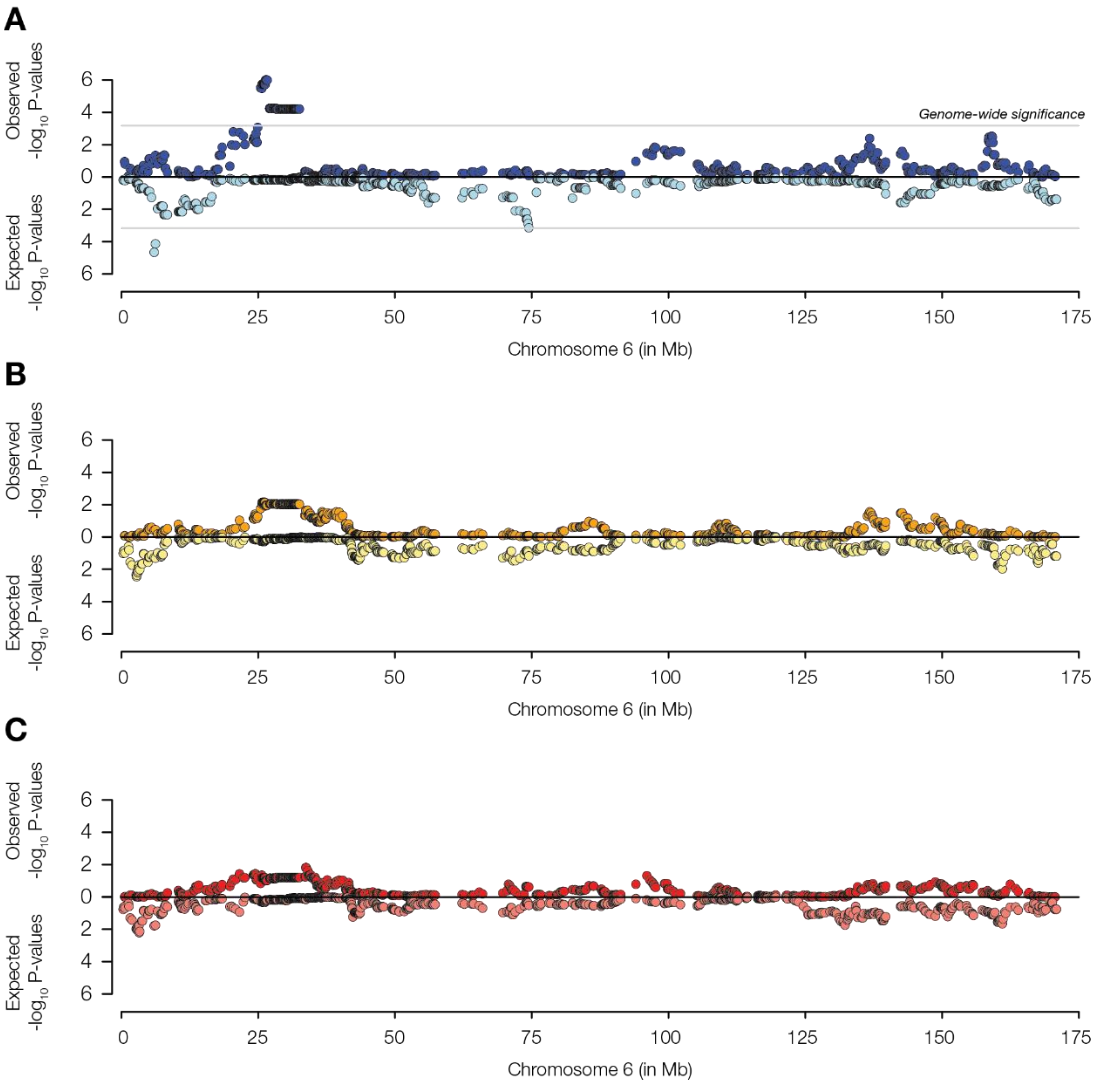

Figure S9. **Observed versus expected ancestry enrichment across the four LA populations studied here for chromosome 6.** Plots show the genome-wide distributions of observed versus expected ancestry enrichment $-\log_{10} P$ values for African (A), European (B), and Native American (C) ancestry components. P-values correspond to the combined cross-population ancestry enrichment values (F_{CS}), calculated as described in the manuscript. Expected values were generated based demographically parameterized simulation of admixed populations, as described in the Methods section. On the African ancestry Manhattan plot, lines indicate genome-wide statistically significant cross-population ancestry enrichment (FDR $q < 0.05$). None of the simulated (expected) data for African ancestry rise to level of significance seen for the observed African ancestry at the MHC locus. There were no genome-wide significant cross-population ancestry enrichment peaks for the European and Native American ancestry components.

Modeling admixture-enabled selection at single loci

A recursive triallelic model of positive selection was used to measure the strength of admixture-enabled selection at the chromosome 6 MHC locus in the Colombia, Mexico, and Peru populations. The model treats alleles as the three ancestry fractions: African, European, and Native American. This model is based on the approach to modelling selection used in the Populus software (https://cbs.umn.edu/populus/overview).

Assuming an African ancestry advantage, the relative fitness (w_{ij}) of a locus with ancestries i and j (ancestry genotypes) is calculated as:

$$w_{ij} = \begin{cases} 1, & if\ i = j = African \\ 1 - sh, & if\ i = African\ or\ j = African \\ 1 - s, & if\ i \neq African\ and\ j \neq African \end{cases}$$

where s is the selection coefficient and h is the dominance coefficient. This can be formulated as a fitness matrix:

		Ancestry j		
		African (A)	European (E)	Native American (N)
Ancestry i	African (A)	1	$1 - (sh)$	$1 - (sh)$
	European (E)	$1 - (sh)$	$1 - s$	$1 - s$
	Native American (N)	$1 - (sh)$	$1 - s$	$1 - s$

For each population, for each ancestry, the ancestry frequency in the next generation ($p_{i,t+1}$) was calculated as:

$$p_{i,t+1} = \frac{p_{i,t} w_i}{\bar{w}}$$

where $p_{i,t}$ is the ancestry frequency (i) in the current generation (t), w_i is the marginal fitness of the ancestry, and $\bar{w}$ is the population mean fitness.

For each row of the fitness matrix, the marginal fitness (w_i) was calculated as

$$w_i = \sum_j w_{ij} p_j$$

where w_{ij} is the relative fitness of the ancestry genotype and w_{ij} is the frequency of the ancestry in the current generation.

For each generation, the population mean fitness ($\bar{w}$) was calculated as:

$$\bar{w} = \sum_i \sum_j w_{ij} p_i p_j$$

where w_{ij} is the relative fitness of the ancestry genotype, p_i is the frequency of the first ancestry, and p_j is the second of the second ancestry.

For each population, the model was run using s from 0-1.0, in increments of 0.001, and $h = 0.05$, until the final African ancestry frequency at 20 generations was the same as that observed for the chromosome 6 haplotype. The selection coefficient at this convergence was taken to be the strength of selection for the given population for the chromosome 6 African enriched haplotype.

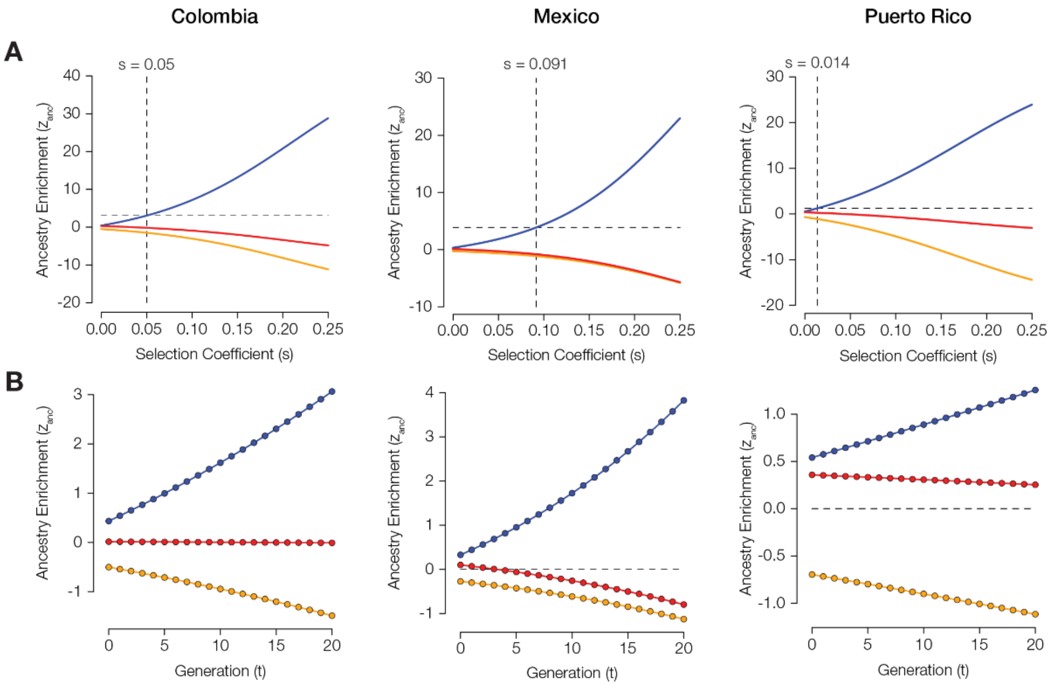

Figure S10. **Modelling the strength of admixture-enabled selection at the MHC locus.** The strength of admixture-enabled selection for African haplotypes at the MHC locus was modelled as described on pages 8-9. The three-ancestry population genetic model was run across a range of positive selection coefficient (s) values to identify the strength of selection needed to explain the observed levels of African ancestry enrichment at the MHC locus in the Colombia, Mexico, and Puerto Rico populations. The top panels for each population show the predicted levels of ancestry enrichment (z_{anc}, y-axis) across a range of different selection coefficients (s, x-axis). The intersection of the observed levels of African ancestry enrichment and their corresponding s-values are indicated with dashed lines. The bottom panels show the trajectory of predicted ancestry enrichment and depletion (z_{anc}, y-axis) over time (t generations, x-axis) for each population given the inferred selection coefficient s for each population.

Table S2. Human populations analyzed as part of this study. Populations are organized into continental groups, for both proxy ancestral reference populations and Latin American populations, and the number of genome (genotype) samples from each population is shown.

Population group	Dataset[1]	Geographical source	*n*	Population group	Dataset[1]	Geographical source	*n*
**African**	1KGP	Americans of African Ancestry in SW USA	61	**Native American**	Reich et al	Waunana in Colombia	3
					Reich et al	Kogi in Colombia	4
	1KGP	Mende in Sierra Leone	85		Reich et al	Mixtec in Mexico	5
	1KGP	African Caribbeans in Barbados	96		Reich et al	Embera in Colombia	5
	1KGP	Esan in Nigeria	99		Reich et al	Guahibo in Colombia	6
	1KGP	Luhya in Webuye, Kenya	99		Reich et al	Ticuna in Brazil	6
	1KGP	Yoruba in Ibadan, Nigeria	108		Reich et al	Guarani in Paraguay	6
	1KGP	Gambian in Western Division in the Gambia	113		Reich et al	Piapoco in Colombia	7
					HGDP	Suruí in Brazil	8
**European**	1KGP	British in England and Scotland	91		Reich et al	Inga in Colombia	9
	1KGP	Finnish in Finland	99		Reich et al	Wayuu in Colombia	11
	1KGP	Utah Residents (CEPH) with Northern and Western European Ancestry	99		Reich et al	Kaqchikel in Guatemala	13
					HGDP	Pima in Mexico	14
	1KGP	Iberian Population in Spain	107		HGDP	Karitiana in Brazil	14
	1KGP	Toscani in Italia	107		Reich et al	Mixe in Mexico	17
**Latin American**	1KGP	Mexican Ancestry in Los Angeles USA	64		HGDP	Maya in Mexico	21
					Reich et al	Aymara in Bolivia	23
	1KGP	Peruvians from Lima, Peru	85		Reich et al	Tepehuano in Mexico	25
	1KGP	Colombians from Medellin, Colombia	94		Reich et al	Quechua in Peru	40
	1KGP	Puerto Ricans from Puerto Rico	104		Reich et al	Zapotec in Mexico	43

[1]1KGP = 1000 Genomes Project; HGDP = Human Genome Diversity Project; Reich et al [1]

Polygenic ancestry enrichment

Polygenic trait gene set curation

Gene sets for polygenic traits were curated from a number of literature and database sources, as shown below, to represent a wide array of phenotypes.

Table S3. Sources of the polygenic trait gene sets analyzed as part of this study. For each polygenic trait gene set source, the number of phenotypes and reference are shown.

Phenotype source	*n*	Reference
NHGRI-EBI GWAS Catalog	306	[2]
InnateDB	128	[3]
Blood Transcription Modules	209	[4]
Blood Informative Transcripts	9	[5]
Innate Immune System	9	[6]
GIANT	7	[7]
Custom gene sets of interest	10	na

Whenever possible, the gene sets were taken directly from the literature or the database. If gene sets were not directly accessible, SNP-level data was collected and directly mapped to genes to generate the gene set. Trait-specific gene sets from the NHGRI-EBI GWAS Catalog [2] were collected from SNP sets that were mapped using EBI's in-house pipeline; only SNPs that fell within a gene and were implicated at a genome-wide significance level of $P \leq 5\times10^{-8}$ with the phenotype were used to generate the gene sets. Each polygenic trait from the Genetic Investigation of ANthropometric Traits (GIANT) consortium [7] had gene sets mapped according to specifications of the individual paper. All remaining gene sets from the literature were mapped using NCBI's dbSNP database as needed. To control for any linkage between genes, linkage disequilibrium (LD) pruning was performed on each gene set using PLINK. For each set, pruning was performed on all gene pairs with genic SNP $r^2 > 0.1$; if this was found to be true then only one member of the gene pair was retained for polygenic ancestry enrichment analysis. After LD pruning was complete, the gene sets were filtered based on size so that all phenotypes included in the analysis contained two or more genes.

Statistical significance of polygenic ancestry enrichment

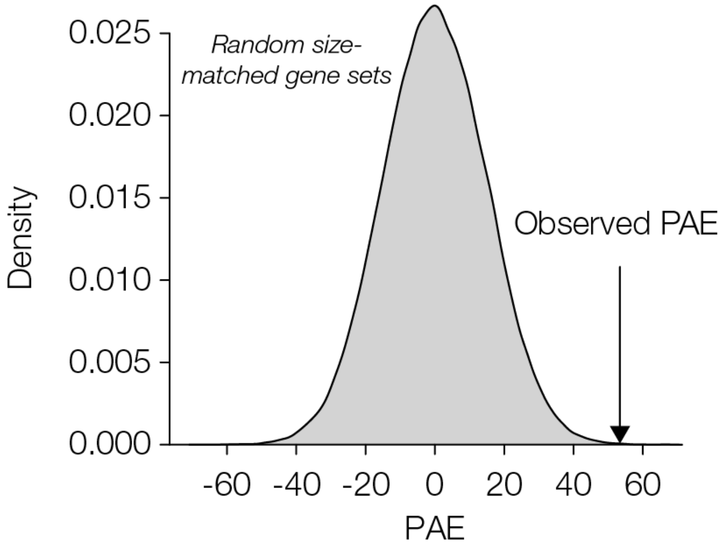

Figure S11. **Simulation of random polygenic trait gene sets used to compute statistical significance of polygenic ancestry enrichment (PAE).** Size-matched random gene sets are simulated to generate an expected (null) distribution of PAE values.

References

1. Reich D, Patterson N, Campbell D, Tandon A, Mazieres S, Ray N, Parra MV, Rojas W, Duque C, Mesa N, et al: **Reconstructing Native American population history.** *Nature* 2012, **488:**370-374.
2. MacArthur J, Bowler E, Cerezo M, Gil L, Hall P, Hastings E, Junkins H, McMahon A, Milano A, Morales J, et al: **The new NHGRI-EBI Catalog of published genome-wide association studies (GWAS Catalog).** *Nucleic Acids Res* 2017, **45:**D896-D901.
3. Li S, Rouphael N, Duraisingham S, Romero-Steiner S, Presnell S, Davis C, Schmidt DS, Johnson SE, Milton A, Rajam G, et al: **Molecular signatures of antibody responses derived from a systems biology study of five human vaccines.** *Nat Immunol* 2014, **15:**195-204.
4. Breuer K, Foroushani AK, Laird MR, Chen C, Sribnaia A, Lo R, Winsor GL, Hancock RE, Brinkman FS, Lynn DJ: **InnateDB: systems biology of innate immunity and beyond--recent updates and continuing curation.** *Nucleic Acids Res* 2013, **41:**D1228-1233.
5. Preininger M, Arafat D, Kim J, Nath AP, Idaghdour Y, Brigham KL, Gibson G: **Blood-informative transcripts define nine common axes of peripheral blood gene expression.** *PLoS Genet* 2013, **9:**e1003362.
6. Deschamps M, Laval G, Fagny M, Itan Y, Abel L, Casanova JL, Patin E, Quintana-Murci L: **Genomic Signatures of Selective Pressures and Introgression from Archaic Hominins at Human Innate Immunity Genes.** *Am J Hum Genet* 2016, **98:**5-21.
7. **GIANT: Genetic Investigation of ANthropometric Traits** [http://portals.broadinstitute.org/collaboration/giant/index.php/GIANT_consortium]
