# Supplementary material for: Admixture-enabled selection for rapid adaptive evolution in the Americas
Source: Genome Biol. 2020 Feb 7;21:29. doi: 10.1186/s13059-020-1946-2 (PMC7006128; doi:10.1186/s13059-020-1946-2)
Supplement: Supplementary file 3 — Additional file 3. Review history. [file 13059_2020_1946_MOESM3_ESM.docx]

Review History

**First round of review**

**Reviewer 1**

**Are you able to assess all statistics in the manuscript, including the appropriateness of statistical tests used?**

Yes

**Comments to author:**

This manuscript analyses the adaptive history of four Latin American (LA) populations from the 1000 Genomes Project. The main objective of the project is not novel, as the same authors have already published a very similar study using the Colombian samples from this dataset. However, they do address one novel interesting question regarding the validity of a strong selective signal at the MHC locus. They also report a novel test that aims to find evidence of polygenic selection post-admixture. Overall, I find the study interesting, but I do not find that the statistical analysis used to validate their results reflect the true uncertainty and complications present in post-admixture selections scans.

My major concern involves their simulations. The authors validated their only observed significant ancestry enrichment by generating a random admixture process parameterized by the observed genome-wide ancestry proportions. This raises some concerns:

1. By simply sampling the ancestry proportions from a genome-wide mean, the authors assume that all the local ancestry assignments are always perfect, which is not always the case.

2. I would rather encourage the authors to use a simulation software in order to properly model admixture, using sensible parameters that reflect the demographic history of Latin American populations as closely as possible (e.g. using a recent time of admixture, admixture proportions as observed in their data, etc).

3. The MHC locus is known to have a distinct recombination rate compared to that of the genome, as well as a high number of polymorphic sites. Given that their main result implicates this locus, I wonder whether using recombination rates that have been estimated at this region (rather than for example a using a constant recombination rate) could affect the accuracy of the local ancestry assignments and cause strong deviations in ancestry.

4. Local ancestry assignment involves using proxy populations instead of the actual populations involved in the admixture. Given that the authors used Native American samples that are not very closely related to the actual Native American samples involved in the process (this would be the case for the samples for Puerto Rico for example), I wonder what would be the effect of using populations that are not closely related in the admixture process on the local ancestry assignments. This could be easily modeled.

5. Lastly, in order to actually simulate the effect of genetic drift, the authors could reduce the effective size of the admixed population after the last admixture event.

My second concern involves their novel polygenic ancestry enrichment test. The authors generated a null distribution of polygenic ancestry enrichment (PAE) values by 10,000 random permutations of size-matched genes. However, the authors do not indicate the exact way they size-matched each gene. Was it using quantiles? Or was it using some other type of threshold? The author should mention this. Also, I wonder whether using the number of SNPs at each gene rather than the size would be a more sensible way to match the genes. Although these are likely to be correlated, the number of SNPs at a gene is the underlying factor that affects the local ancestry assignment. Lastly, why not also matched them by other genomic factors, for example by similar values of recombination rates? I assume that this factor might also affect the ancestry assignments and if so, might also be necessary to account for it.

Minor comments:

On Figure 2, it seems that the Peruvian sample is actually showing an increase of Native American ancestry at the MHC locus, which is not observed in the other simulations. The authors should address this apparent inconsistency in their results/discussion section.

To select SNPs with significant iHS scores the authors use an absolute value higher than 2 as a threshold. Does this value represents an actual outlier score (i.e. top 1% or top 0.5%) in their genome-wide distribution?

**Reviewer 2**

**Are you able to assess all statistics in the manuscript, including the appropriateness of statistical tests used?**

No, I do not feel adequately qualified to assess the statistics

**Comments to author:**

This is a well-written manuscript in an area of research that will be of wide interest to a multi-disciplinary audience. It brings a sophisticated analytical approach to a topic that has been attempted by others with limited success. The study of the effects of admixture, and of the adaptive potential for admixed populations, is of continuing interest to geneticists, human biologists, anthropologists, and historians, especially in the Americas. This paper is a solid contribution to this literature.

Three innovations employed here are especially noteworthy: 1) use of genomic scale data in multiple admixed populations of Latin America and the Afro-Caribbean, 2) correction for multiple hypothesis tests in genomic screens for selection in admixed populations, and 3) the use of admixture simulation as a check on the formal analyses. The latter approach is particularly gratifying in demonstrating that the cross-population approach for the single locus analyses was powered sufficiently to detect selection given the sample sizes.

I am not expert is all the analytical methods employed, but do have some minor suggestions for revision that will strengthen the manuscript.

1. Provide a reference for the Benjamini-Hochberg method employed to control for multiple tests.

2. Given the long history of attributing the demographic collapse of Native American populations to the introduction of new diseases, some elaboration on the role of the three pathways involved in viral immune response described on p. 10 (lines 175-180) would be helpful and expand the scope of interest for readers.

3. I would suggest not using the term 'pre-evolved' adaptive utility (e.g., top of p. 11). The term by itself can be confusing, even misleading. Although the discussion, and explanation, of this in the last paragraph of that page is clear, I would suggest avoiding the term.

**Authors Response**

**Point-by-point responses to the reviewers’ comments:**

Responses to reviewers for “Admixture-enabled selection for rapid adaptive evolution in the Americas”

Reviewer reports:

Reviewer #1:

This manuscript analyses the adaptive history of four Latin American (LA) populations from the 1000 Genomes Project. The main objective of the project is not novel, as the same authors have already published a very similar study using the Colombian samples from this dataset. However, they do address one novel interesting question regarding the validity of a strong selective signal at the MHC locus. They also report a novel test that aims to find evidence of polygenic selection post-admixture. Overall, I find the study interesting, but I do not find that the statistical analysis used to validate their results reflect the true uncertainty and complications present in post-admixture selections scans.

*Response: We appreciate the reviewer’s suggestions for several additional controls to support our findings. We have executed all the controls, as described below in detail for each comment, and modified the manuscript accordingly.*

My major concern involves their simulations. The authors validated their only observed significant ancestry enrichment by generating a random admixture process parameterized by the observed genome-wide ancestry proportions. This raises some concerns:

1. By simply sampling the ancestry proportions from a genome-wide mean, the authors assume that all the local ancestry assignments are always perfect, which is not always the case.

*Response: This is absolutely correct. We use a very stringent local ancestry assignment confidence threshold of 0.98 with RFMix to deal with this issue. We have added this information to the revised manuscript (see lines 299-300). It is also worth noting there that the local ancestry assignments generated with RFMix yield global ancestry estimates that are very highly correlated with estimates from ADMIXTURE, further supporting their accuracy (see Figure S1 in Additional file 1). As described below, we have performed additional controls to provide support for the local ancestry assignments.*

2. I would rather encourage the authors to use a simulation software in order to properly model admixture, using sensible parameters that reflect the demographic history of Latin American populations as closely as possible (e.g. using a recent time of admixture, admixture proportions as observed in their data, etc).

*Response: As suggested by the reviewer, we have used a simulation software to properly simulate admixture, using parameters that reflect the known history of Latin American populations as closely as possible. We used the “Admixture simulation tool” developed by Mark Wright when he was a member of the Carlos Bustamante laboratory. The package can be found at https://github.com/slowkoni/admixture-simulation We chose this simulation software because (1) it was developed by one of the world’s leading laboratories for genetic ancestry and admixture research, (2) it appeared to be very well maintained and supported, consistent with reliable development practices, (3) it was developed in conjunction with and is released together with RFMix version 2 (https://github.com/slowkoni/rfmix), (4) it installs and runs efficiently, and (5) it allows for the specification of demographic parameters. It should be noted that we tried several other admixture simulation programs, including SLIM, none of which worked reliably for us.*

*We ran the demographically parameterized simulation for chromosome 6 in light of the significant results that we observed there and the anomalous patterns of recombination seen for this chromosome, especially for the MHC locus (see reviewer comment #3 below). The results of this parameterized admixture simulation are similar to what we saw for our original simulation. Most importantly, the expected levels of African ancestry enrichment across chromosome 6, and at the MHC locus in particular, do not rise to levels that we observe for the LA populations studied here. Furthermore, the simulation does not provide any evidence of spurious ancestry enrichment in the European and Native American ancestry components. These new simulation results further support the conservative nature of our cross-population approach to the delineation of ancestry enrichment. We show these as results as Figure S9 in the revised Additional file 1. We describe how the simulation was parameterized and run in the Methods section (see lines 333-344) and described the new findings in the results section (see lines 134-140 and 147-151). We used a conservative set of parameters in terms of the effective population size and the number of generations to admixture in an effort to simulate the most extreme bottlenecks that could be expected to yield the highest variance in local ancestry fractions by chance alone.*

3. The MHC locus is known to have a distinct recombination rate compared to that of the genome, as well as a high number of polymorphic sites. Given that their main result implicates this locus, I wonder whether using recombination rates that have been estimated at this region (rather than for example a using a constant recombination rate) could affect the accuracy of the local ancestry assignments and cause strong deviations in ancestry.

*Response: As suggested by the reviewer, we re-ran RFMix using a constant recombination rate, as opposed to the recombination rates estimated from the 1KGP, and compared the local ancestry assignment results for each run of RFMix (variable versus constant recombination rate). The results generated via the two approaches are highly concordant (r=0.99) as can be seen in the new Figure S2 (briefly discussed in lines 95-97). Taken together with the high RFMix accuracy threshold we use (see response to question #1 above), these new results indicate that the deviations from local ancestry at the MHC locus are not due to the recombination rate. We also elaborate on how local ancestry assignments are particularly robust for the kinds of very distinct continental ancestries analyzed in our paper in response to question #4 below.*

4. Local ancestry assignment involves using proxy populations instead of the actual populations involved in the admixture. Given that the authors used Native American samples that are not very closely related to the actual Native American samples involved in the process (this would be the case for the samples for Puerto Rico for example), I wonder what would be the effect of using populations that are not closely related in the admixture process on the local ancestry assignments. This could be easily modeled.

*Response #4: For this study, we are assigning local ancestry for continental ancestry groups that are very diverged – African, European, and Native American. Thus, it should not matter if the exact same Native American reference populations are not used to infer local ancestry since the Native American segments in the admixed populations will be far more closely related to reference Native American populations that to reference African or European populations. As suggested by the reviewer, we checked this by using East Asian reference populations (CHB and JPT) as a surrogate for Native American reference populations and re-ran RFMix. The original Native American ancestry assignments for RFMix run with Native American reference populations are highly correlated (r=0.99) with the Native American ancestry assignments inferred with RFMix re-run with East Asian reference populations (see Figure S3 and accompanying text in Additional file 1 and revised lines 95-97).*

5. Lastly, in order to actually simulate the effect of genetic drift, the authors could reduce the effective size of the admixed population after the last admixture event.

*Response: We did this as suggested using the new admixture simulation software (see response to question #2 above).*

My second concern involves their novel polygenic ancestry enrichment test. The authors generated a null distribution of polygenic ancestry enrichment (PAE) values by 10,000 random permutations of size-matched genes. However, the authors do not indicate the exact way they size-matched each gene. Was it using quantiles? Or was it using some other type of threshold? The author should mention this. Also, I wonder whether using the number of SNPs at each gene rather than the size would be a more sensible way to match the genes. Although these are likely to be correlated, the number of SNPs at a gene is the underlying factor that affects the local ancestry assignment. Lastly, why not also matched them by other genomic factors, for example by similar values of recombination rates? I assume that this factor might also affect the ancestry assignments and if so, might also be necessary to account for it.

*Response: We have clarified what we meant by “size matched gene sets” in the original manuscript. We were not referring to the size of genes with respect to their length; rather, we were referring to the number of genes in each permuted set. In the revised manuscript, we write “… 10,000 random permutations of gene sets, each of which is made up of the same number of genes as the trait-specific gene set being compared…” (see lines 357-358). It should be noted here that the size of the genes, i.e. their length, is not important since the length of the ancestry-specific haplotype assignments tend to be much longer than genes, given the fact that admixture occurred within the last 20 generations in the Americas. Accordingly, the vast majority of genes fall within a single local ancestry block, so it will not matter what the length of genes used in the random permutation is. The same thing holds for the local recombination rate since the size of the ancestry blocks is determined by the amount of recombination that has occurred since admixture. Again, the important thing is that the genes fall within single ancestry blocks.*

Minor comments:

On Figure 2, it seems that the Peruvian sample is actually showing an increase of Native American ancestry at the MHC locus, which is not observed in the other simulations. The authors should address this apparent inconsistency in their results/discussion section.

*Response: Peru shows higher European ancestry at this locus, but it is not statistically significant in any of our tests (see Figure 2).*

To select SNPs with significant iHS scores the authors use an absolute value higher than 2 as a threshold. Does this value represents an actual outlier score (i.e. top 1% or top 0.5%) in their genome-wide distribution?

*Response: We changed this threshold to iHS > 2.5 as suggested in the original paper where iHS was developed (ref 21 in the manuscript and PMID: 16494531). The iHS threshold of 2.5 corresponds to the top 1.4 percent of values in the data analyzed here. These changes are shown on lines 155-157 of the revised manuscript and in revised Figures 2 and 3.*

Reviewer #2:

This is a well-written manuscript in an area of research that will be of wide interest to a multi-disciplinary audience. It brings a sophisticated analytical approach to a topic that has been attempted by others with limited success. The study of the effects of admixture, and of the adaptive potential for admixed populations, is of continuing interest to geneticists, human biologists, anthropologists, and historians, especially in the Americas. This paper is a solid contribution to this literature.

Three innovations employed here are especially noteworthy: 1) use of genomic scale data in multiple admixed populations of Latin America and the Afro-Caribbean, 2) correction for multiple hypothesis tests in genomic screens for selection in admixed populations, and 3) the use of admixture simulation as a check on the formal analyses. The latter approach is particularly gratifying in demonstrating that the cross-population approach for the single locus analyses was powered sufficiently to detect selection given the sample sizes.

I am not expert is all the analytical methods employed, but do have some minor suggestions for revision that will strengthen the manuscript

1. Provide a reference for the Benjamini-Hochberg method employed to control for multiple tests.

*Response: As suggested, we provide a reference for the Benjamini-Hochberg method employed to control for multiple tests (reference 43 cited on line 319).*

2. Given the long history of attributing the demographic collapse of Native American populations to the introduction of new diseases, some elaboration on the role of the three pathways involved in viral immune response described on p. 10 (lines 175-180) would be helpful and expand the scope of interest for readers.

*Response: As suggested, we have added a new section entitled “Admixture-enabled selection and the immune system” to the Discussion in the revised manuscript to discuss the implications of these results (see lines 244-265).*

3. I would suggest not using the term 'pre-evolved' adaptive utility (e.g., top of p. 11). The term by itself can be confusing, even misleading. Although the discussion, and explanation, of this in the last paragraph of that page is clear, I would suggest avoiding the term.

*Response: We have removed the term ‘pre-evolved’ adaptive utility as suggested and expanded slightly on how we explain the concept (see lines 200-201).*

**Second round of review**

**Reviewer 1**

This is a resubmission of a manuscript describing selection post-admixture in four different Latin American populations. As noted in my previous review, the authors address one novel questions regarding the validity of a strong selection signal at the MHC locus. The authors have done a good job addressing the concerns of reviewers, and particularly my concern regarding the accuracy of their local ancestry estimations. However, I still have a few concerns, which I believe can be easily addressed.

My first concern is that the authors showed that their RFMix results are robust when using either a constant recombination rate, or the recombination rates estimated from the 1KG Project recombination map. However, I still wonder whether the specific recombination rates at the MHC locus might be causing the strong deviations. Given that the authors have already developed a simulation-based pipeline, I would like to see the results of their simulations, using recombination rates estimated at the MHC locus, and also using the same SNP density.

Second, as the authors points out in their response, ‘the vast majority of genes fall within a single local ancestry block’, and therefore I wonder how much of the polygenic selection signal could be driven by clusters of genes that happen to lie within a given local ancestry block.

Minor concerns:

- At line 132, there seems to be a line break, which produces an incomplete sentence.

**Reviewer 2**

All my suggestions in prior review have been effectively addressed.

**Author’s response**

**Point-by-point response to reviewers comments:**

Reviewer #1: This is a resubmission of a manuscript describing selection post-admixture in four different Latin American populations. As noted in my previous review, the authors address one novel questions regarding the validity of a strong selection signal at the MHC locus. The authors have done a good job addressing the concerns of reviewers, and particularly my concern regarding the accuracy of their local ancestry estimations. However, I still have a few concerns, which I believe can be easily addressed.

Comment #1: My first concern is that the authors showed that their RFMix results are robust when using either a constant recombination rate, or the recombination rates estimated from the 1KG Project recombination map. However, I still wonder whether the specific recombination rates at the MHC locus might be causing the strong deviations.

*Below, we show that the specific recombination rates at the MHC locus do not cause ancestry deviations. The table shows the number of ancestry assignments at the MHC locus for each possible combination of two haplotpyes at the locus: AFR-African, EUR-European, NAT-Native American. Please note that this results is essentially the same as what was previously shown in response to the reviewer’s first round of suggestions (see Supplementary Figure S2).*

|  |  | **Uniform Recombination Rate** | | | | | |
| --- | --- | --- | --- | --- | --- | --- | --- |
|  |  | **AFR, AFR** | **AFR, EUR** | **AFR, NAT** | **EUR, EUR** | **EUR, NAT** | **NAT, NAT** |
| **Inferred Recombination Rate** | **AFR, AFR** | 339 | 0 | 0 | 0 | 0 | 0 |
|  | **AFR, EUR** | 1 | 25 | 0 | 1 | 0 | 0 |
|  | **AFR, NAT** | 0 | 0 | 10 | 0 | 0 | 0 |
|  | **EUR, EUR** | 0 | 4 | 0 | 215 | 0 | 0 |
|  | **EUR, NAT** | 0 | 1 | 0 | 0 | 57 | 0 |
|  | **NAT, NAT** | 0 | 0 | 0 | 0 | 0 | 181 |

Comment #2: Given that the authors have already developed a simulation-based pipeline, I would like to see the results of their simulations, using recombination rates estimated at the MHC locus, and also using the same SNP density.

*As requested, below are the results of the simulations, using recombination rates estimated at the MCH locus and also using the same SNP density. The expected levels of African ancestry enrichment are far lower than the observed levels. Please note that this results is essentially the same as what was previously shown in response to the reviewer’s first round of suggestions (see Supplementary Figure S9).*

**Comment #3:** Second, as the authors points out in their response, ‘the vast majority of genes fall within a single local ancestry block’, and therefore I wonder how much of the polygenic selection signal could be driven by clusters of genes that happen to lie within a given local ancestry block.

*The polygenic selection signal cannot be driven by clusters of genes because we used linkage disequilibrium (LD) pruning on the gene sets used to screen for polygenic selection. The LD pruning control is described in the Methods section on “Polygenic ancestry enrichment” pg. 18 “All gene sets were LD pruned with a threshold of r2 > 0.1 using PLINK.”*

Minor concerns:

- At line 132, there seems to be a line break, which produces an incomplete sentence.

*We corrected this.*
